# Association of entirely claims-based frailty indices with long-term outcomes in patients with acute myocardial infarction, heart failure, or pneumonia: a nationwide cohort study in Turkey

**DOI:** 10.1016/j.lanepe.2021.100183

**Published:** 2021-07-29

**Authors:** Harun Kundi, Nazim Coskun, Metin Yesiltepe

**Affiliations:** a Department of Cardiology, Ankara City Hospital, Ankara, Turkey; b Department of Digital Hospital and Analytical Management Unit, Ankara City Hospital, Ankara, Turkey; c Department of Nuclear Medicine, Ankara City Hospital, Ankara, Turkey; d Department of Pharmacology, Ankara City Hospital, Ankara, Turkey; e Department of Pharmacology, Physiology & Neuroscience, New Jersey Medical School, Rutgers The State University of New Jersey, NJ, USA

**Keywords:** AMI, Heart Failure, Pneumonia, Mortality, Readmission, Frailty

## Abstract

**Background:**

Several countries have increasingly focused on improving care for acute myocardial infarction (AMI), heart failure (HF), and pneumonia to reduce their readmissions and mortality rates. Frailty is becoming increasingly important to accurately predict healthcare utilization for the aging population. The preferred method for the measurement of frailty remains unclear, and current risk-adjustment models do not account for frailty. We sought to compare commonly used frailty indices in terms of the ability to predict clinical adverse outcomes in AMI, HF, and pneumonia patients.

**Methods:**

A nationwide cohort study included AMI, HF, and pneumonia with 65 years and older patients in the Turkey between January 1 and December 31, 2018. The primary predictor of interest was frailty. We used two claims-based frailty indices (Johns Hopkins Claims-Based Frailty Index and Hospital Frailty Risk Score) to assess frailty. The main outcome was all-cause long-term mortality up to 3 years. Time to death was calculated as the time period between the date of first admission and the date of death. Patients were censored as of September 30, 2020, which marked the end of the follow-up period.

**Findings:**

Of the 200,948 patients, 35,096 (17.5%) had AMI, 62,403 (31.1%) had HF, and 103,449 (51.5%) had pneumonia. Johns Hopkins Claims-Based Frailty Index (c-statistics for long-term mortality: 0.68 in AMI, 0.61 in HF, 0.64 in pneumonia) was better compared to Hospital Frailty Risk Score (c-statistics for long-term mortality: AMI=0.62, HF=0.58, pneumonia=0.62) (DeLong p<0.001 in all).

**Interpretation:**

Readmission and mortality rates after AMI, HF, and pneumonia gradually increases with increasing frailty score. While the Hospital Frailty Risk Score had a better discrimination for predicting readmissions, Johns Hopkins Claims-Based Frailty Index had a better discrimination for predicting mortality. These findings should be taken into account for a better evaluation of hospital performance.

**Funding:**

This study was supported by funding from The Scientific and Technological Research Council of Turkey (grant 120S422, HK).

**Research in context**

**Evidence before this study**

Many healthcare systems financially penalize or award hospitals based on their relative performance on adverse outcomes according to their risk-adjustment models in acute myocardial infarction (AMI), heart failure (HF), and pneumonia. It is already known that frailty, a syndrome defined as a decline in biologic reserve and an increased vulnerability, is a critical indicator of patient complexity that contributes to the risk of adverse outcomes in several medical conditions. In addition, it is already known that the addition of frailty measurement to traditional comorbidity-based risk-prediction models improve the prediction of outcomes for AMI, HF, and pneumonia. However, the preferred method for the measurement of frailty remains unclear, and current risk-adjustment models do not account for frailty.

**Added value of this study**

Administrative International Statistical Classification of Diseases, Clinical Modification, 10^th^ Revision codes can be used to evaluate frailty. Readmission and mortality rates after AMI, HF, and pneumonia gradually increases with an increasing frailty score. While the Hospital Frailty Risk Score had a better discrimination for predicting readmissions, Johns Hopkins Claims-Based Frailty Index had a better discrimination for predicting mortality.

**Implications of all the available evidence**

These findings should be taken into account for a better evaluation of hospital performance by countries.

Alt-text: Unlabelled box

## 1 Introduction

Understanding the association between adverse outcomes and frailty, a syndrome defined as a decline in biologic reserve and an increased vulnerability, is becoming increasingly important to accurately predict healthcare utilization for the aging population [1,2]. National guidelines strongly recommend an objective evaluation of frailty to optimize patient selection [3,4]. However, the range of available measures raises issues with consistency. Different prevalence estimations and effect sizes have been reported in previous studies [5]. Therefore, frailty is not routinely measured and has not been widely implemented in clinical practice. Administrative claims constitute an alternative source of data by which frailty might be more easily assessed. Evaluation of frailty using electronic health records, a method which does not require additional workload or data collection, has become more prominent in recent years [6].

In the past decade, several countries have increasingly focused on improving care for acute myocardial infarction (AMI), heart failure (HF), and pneumonia, which are among the top cause of hospitalization, to reduce their readmissions and mortality rates [7], [8], [9]. Many healthcare systems financially penalize or award hospitals based on their relative performance on adverse outcomes according to their risk-adjustment models in these medical conditions [10], [11], [12], [13]. We previously showed that frailty is a critical indicator of patient complexity that contributes to the risk of adverse outcomes in several medical conditions [14], [15], [16]. However, the preferred method for the measurement of frailty remains unclear, and current risk-adjustment models do not account for frailty.

In the current study, first, we sought to validate two commonly used frailty indices based entirely on International Statistical Classification of Diseases, Clinical Modification (ICD-CM) Codes in acute myocardial infarction (AMI), heart failure (HF), and pneumonia patients. Second, we sought to compare commonly used frailty indices derived from routinely collected administrative data in terms of their ability to predict clinical adverse outcomes.

## 2 Methods

### 2.1 Study cohort and clinical covariates

All hospitalized patients older than 65 years of age with a principal discharge diagnosis of AMI, HF, or pneumonia (eTable 1) between January 1, 2018, and December 31, 2018 in Turkey, were planned to be included in the study. All data were obtained from national digital health record systems, including the “Public Health Management System module” and the “e-Pulse” system to obtain patients characteristics, ICD-10-CM diagnosis codes in the last two years, as explained in detail in previous reports [16], [17], [18]. All study data were recalled from the national database maintained by the Republic of Turkey Ministry of Health. The first index hospitalization was selected for patients with multiple hospitalizations within the study period. Patients admitted for AMI and then discharged on the same day were excluded as they were unlikely to be clinically significant AMIs.

Baseline covariates were ascertained using secondary diagnosis codes, as well as from all principal and secondary diagnosis codes from all hospitalizations in 2-years period prior to the date of admission for the index hospitalization (eTable 2).

### 2.2 Assessment of frailty indices

The primary predictor of interest was frailty. We used two claims-based frailty indices to assess frailty.

First, we used Hospital Frailty Risk Score, an index which was previously developed and validated in an older British cohort [19]. It has been also externally validated in frail elder patients from Canada and US, where it was found to be independently associated with short-term adverse outcomes. The calculation of Hospital Frailty Risk Score was based on one or more of 109 ICD-10-CM diagnosis codes (eTable 3) recalled from all diagnosis codes of any hospitalization within the last two years. According to calculated Hospital Frailty Risk Score, patients were classified into three frailty risk groups based on previously validated cut points as low-risk (<5 points), intermediate-risk (5-15 points), and high-risk (>15 points) [19].

Second, we used Johns Hopkins Claims-based Frailty Index [20] which includes 21 criteria identifiable in claims data, such as demographic variables and markers of physical and cognitive dysfunction, to identify patients meeting Fried's Frailty Phenotype [1]. Since this index was derived from ICD-9-CM diagnostic codes, we appropriately transformed all ICD-9 codes to ICD-10 codes (eTable 4). This index has been extensively validated and shown to predict poor health outcomes including incidence of falls, worsening mobility, hospitalization, and death [15,[21], [22], [23]]. Based on the Johns Hopkins Claims-based Frailty Index algorithm, a score cutoff of ≥ 0.12 was used to identify intermediate-risk frail patients, additionally, and a score cutoff ≥ 0.20 was used to identify high-risk frail patients (eTable 5) [20].

### 2.3 Study outcomes

This study has primarily focused on three outcomes for target populations. The first one was all-cause long-term mortality, determined through a linkage of the national death reporting system, which includes all official Turkish Citizenship deaths. Death Reporting System can provide data exchange between the relevant units of the Republic of Turkey Ministry of Health, Interior Ministry Directorate General of Population and Citizenship Affairs, and Turkish Statistical Institute in order to compile death statistics in a complete, fast, and higher quality manner. It is a web application that can be managed in a single database and in a corporate hierarchical structure. This database includes all official deaths within country borders. Time to death was calculated as the time period between the date of first admission and the date of death. Patients were censored as of September 30, 2020, which marked the end of the follow-up period for all-cause mortality. Additionally, we focused on 1-year all-cause readmission (first subsequent hospitalization within one year) and 1-year all-cause mortality. Patients were censored as of 31 December 2019, which marked the end of the follow-up period for readmission. Since our follow-up period for all-cause long-term mortality was up to September 30, 2020, some of patients could have been lost due to COVID-19. We, therefore, conducted sensitivity analyses to avoid possible influences of pandemic on the mortality rates. For sensitivity analyses, patients who were alived at March 15, 2020, which marked the date of first reported COVID-19 death in Turkey, was defined as time zero. In sensitivity analyses, patients were censored as of September 30, 2020, which marked the end of the follow-up period for all-cause mortality.

### 2.4 Statistical analysis

Continuous and categorical variables are presented as mean (SD) and count (percentage), respectively. We compared all outcomes among frailty categories defined as low-risk, intermediate-risk, and high-risk using the Pearson χ2 or analysis of variance tests as appropriate. Weighted kappa analyses were used to show agreement between two frailty categories for each condition. As sensitivity analyses, scatter plots and spearmen correlation coefficient values were presented according to the two frailty indices (as continuous) for each condition. We constructed multivariable logistic regression models, adjusted for age, sex, and covariates to assess the independent association of levels of frailty with 1-year readmission and mortality. Unadjusted cumulative incidence curves were created to plot long-term all-cause mortality, stratified by frailty categories. To examine the relationship between all-cause long-term mortality and frailty categories, we used multivariable (adjusted for age, sex, and covariates) Cox proportional hazard models. For each outcome, Harrell's concordance statistics (c-statistics) were used to assess models (using only frailty index as continuous) discrimination, and the difference in discrimination between frailty indices was evaluated by the DeLong test [24]. All statistical analyses were performed in Stata version 15.1 (Stata Corporation, College Station, TX, USA). Statistical significance was defined as a p-value of less than 0.05.

**Role of the Funding Source:** The funder had no role in the design and conduct of the study; collection, management, analysis, interpretation of the data and writing of the report.

## 3 Results

### 3.1 Overall results

In total, 200,948 patients (35,096 AMI patients [17.5%], 62,403 HF patients [31.1%], and 103,449 pneumonia patients [51.5%]) were included in analysis. The mean (SD) age of the patients in the study was 74 (7.3) years for patients with AMI, 78 (7.6) years for patients with HF, and 77 (7.9) for patients with pneumonia. Male accounted for 52.9% (n = 18,567) of the admissions for AMI, 45.9% (n = 28,622) of the admissions for HF, and 50.9% (n = 52,683) of the admissions for pneumonia. Overall, history of hypertension (n=94,636, 47.1%) was the most common covariate in each condition. Further information regarding characteristics and covariates of patients are presented in Table 1.

**Table 1:** Baseline demographics, covariates and frailty indices of the study population according to AMI, HF and pneumonia patients

	Acute Myocardial Infarction	Heart Failure	Pneumonia
	n=35,096	n=62,403	n=103,449
**Age, mean (SD)**	74.91 (7.27)	77.88 (7.58)	77.40 (7.79)
**Male**	18,567 (52.9%)	28,622 (45.9%)	52,683 (50.9%)
**Covariates**			
History of Myocardial Infarction	11,171 (31.8%)	17,554 (28.1%)	12,618 (12.2%)
History of Coronary Artery Bypass Greft	1,832 (5.2%)	3,955 (6.3%)	1,500 (1.4%)
History of Valvular Disease	743 (2.1%)	3,449 (5.5%)	1,395 (1.3%)
Hypertension	18,360 (52.3%)	38,269 (61.3%)	38,007 (36.7%)
Peripheral Vascular Disease	4,750 (13.5%)	9,200 (14.7%)	7,340 (7.1%)
Cerebrovascular Disease	4,024 (11.5%)	9,410 (15.1%)	13,662 (13.2%)
Chronic Obstructive Pulmonary Disease	5,365 (15.3%)	24,273 (38.9%)	53,952 (52.2%)
Diabetes Mellitus	4,071 (11.6%)	11,452 (18.4%)	13,752 (13.3%)
Obesity	670 (1.9%)	3,680 (5.9%)	3,730 (3.6%)
Liver Disease	227 (0.6%)	1,007 (1.6%)	1,025 (1.0%)
Renal Failure	2,000 (5.7%)	8,561 (13.7%)	7,657 (7.4%)
Deficiency Anemia	737 (2.1%)	2,353 (3.8%)	3,734 (3.6%)
Rheumatoid Disease	102 (0.3%)	404 (0.6%)	824 (0.8%)
Peptic Ulcer Disease	3,584 (10.2%)	4,805 (7.7%)	7,478 (7.2%)
Dementia	751 (2.1%)	3,921 (6.3%)	8,748 (8.5%)
Depression	260 (0.7%)	1,453 (2.3%)	1,988 (1.9%)
Cancer	1,082 (3.1%)	2,680 (4.3%)	8,561 (8.3%)
Substance Abuse	7 (0.0%)	13 (0.0%)	28 (0.0%)
Acquired immunodeficiency syndrome	0 (0.0%)	5 (0.0%)	10 (0.0%)
**Johns Hopkins Claims-Based Frailty Index, mean (SD)**	0.10 (0.07)	0.14 (0.09)	0.12 (0.09)
**Johns Hopkins Claims-Based Frailty Index Categories**			
Low-Risk (<0.12)	24,509 (69.8%)	30,622 (49.1%)	60,619 (58.6%)
Intermediate-Risk (0.12-0.20)	6,667 (19.0%)	16,895 (27.0%)	24,320 (23.4%)
High-Risk (>0.20)	3,920 (11,2%)	14,886 (23.9%)	18,600 (18.0%)
**Hospital Frailty Risk Score, mean (SD)**	2.13 (3.52)	3.66 (4.45)	4.02 (4.37)
**Hospital Frailty Risk Score Categories**			
Low-Risk (<5)	30,083 (85.7%)	45,995 (73,7%)	75,498 (72.3%)
Intermediate-Risk (5-15)	4,561 (13.0%)	14,513 (23.3%)	24,499 (23.7%)
High-Risk (>15)	452 (1.3%)	1,895 (3.0%)	3,452 (3.3%)

### 3.2 Frailty indices

The Hospital Frailty Risk Score ranged from 0 to 46. The mean (SD) Hospital Frailty Risk Score was 2.1 (3.5) for patients with AMI, 3.7 (4.5) for patients with HF, and 4.0 (4.4) for patients with pneumonia. In the AMI, HF, and pneumonia populations, respectively, there were 30,083 (85.7%), 45,995 (73,7%) and 75,498 (72.3%) patients defined as low-risk (score <5), 4,561 (13.0%), 14,513 (23.3%) and 24,499 (23.7%) defined as intermediate-risk (score 5–15), and 452 (1.3%), 1,895 (3.0%) and 3,452 (3.3%) defined as high-risk (score >15). (Table 1).

Johns Hopkins Claims-Based Frailty Index ranged from 0 to 1. The mean (SD) Johns Hopkins Claims-Based Frailty Index was 0.1 (0.07) for patients with AMI, 0.14 (0.09) for patients with HF, and 0.12 (0.09) for patients with pneumonia*.* In the AMI, HF, and pneumonia populations, respectively, there were 24,509 (69.8%), 30,622 (49.1%) and 60,619 (58.6%) patients defined as low-risk (score <0.12), 6,667 (19.0%), 16,895 (27.0%) and 24,320 (23.4%) defined as intermediate-risk (score 0.12–0.20), and 3,920 (11,2%), 14,886 (23.9%) and 18,600 (18.0%) defined as high-risk (score >0.20). (Table 1).

**Fig. 1:**
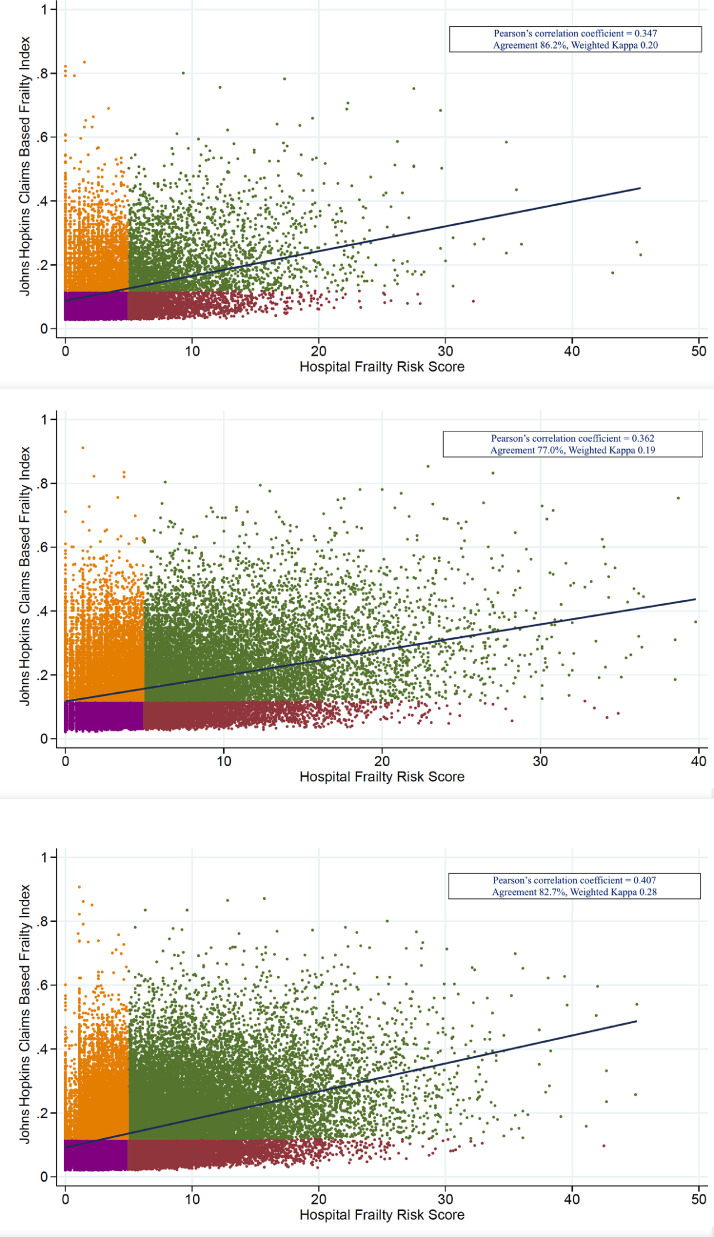
(Purple points: non-frail (low-risk) in both indices, Orange points: frail (intermediate or high-risk) in only Johns Hopkins Claims-Based Frailty, Maroon points: frail (intermediate or high-risk) in only Hospital Frailty Risk Score, Green points: frail (intermediate or high-risk) in both indices) **A.** Scatter plot of Johns Hopkins Claims Based Frailty Index and Hospital Frailty Score in AMI patients **B.** Scatter plot of Johns Hopkins Claims Based Frailty Index and Hospital Frailty Score in HF patients **C.** Scatter plot of Johns Hopkins Claims Based Frailty Index and Hospital Frailty Score in pneumonia patients

There was a modest agreement between Hospital Frailty Risk Score and Johns Hopkins Claims-Based Frailty Index in the AMI (Pearson correlation coefficient = 0.347, 86.2% Agreement, Weighted kappa = 0.20), HF (Pearson correlation coefficient = 0.362, 77.0% Agreement, Weighted kappa = 0.19), and pneumonia (Pearson correlation coefficient = 0.407, 82.7% Agreement, Weighted kappa = 0.28) populations. (Fig. 1)

### 3.3 Outcomes

**Fig. 2:**
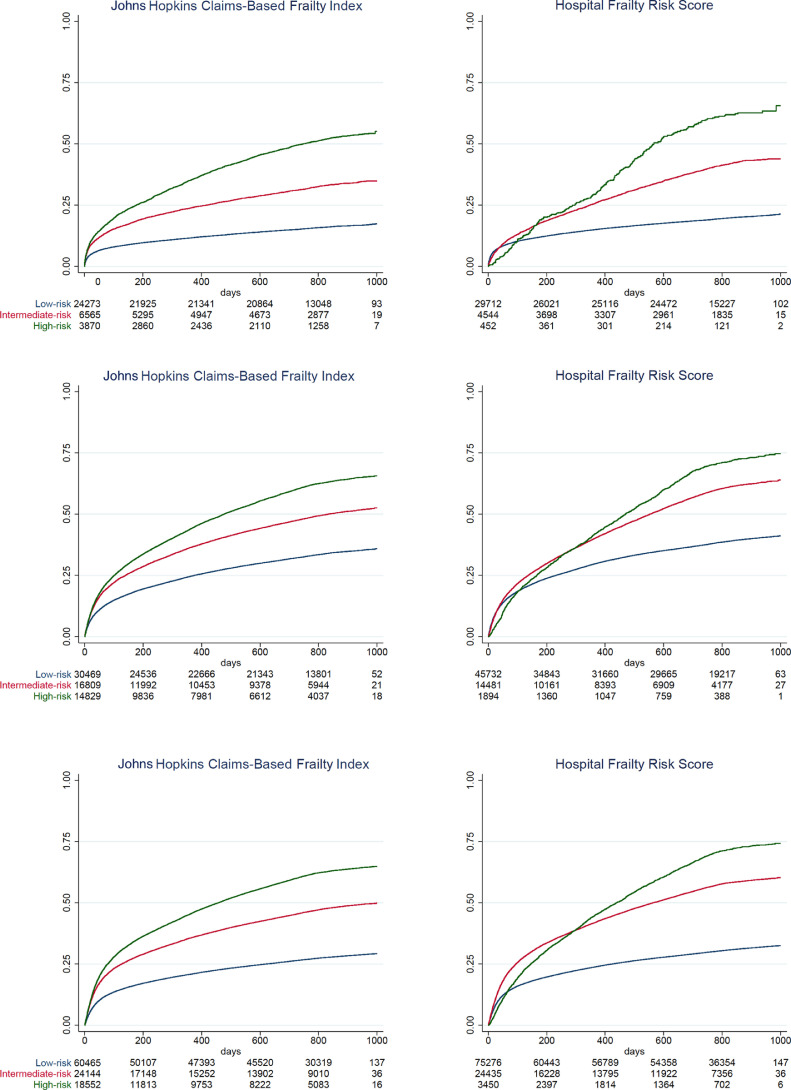
**A.** Kaplan–Meier long-term all-cause mortality curves according to Johns Hopkins Claims-Based Frailty and Hospital Frailty Risk Score categories in AMI patients **B.** Kaplan–Meier long-term all-cause mortality curves according to Johns Hopkins Claims-Based Frailty and Hospital Frailty Risk Score categories in HF patients **C.** Kaplan–Meier long-term all-cause mortality curves according to Johns Hopkins Claims-Based Frailty and Hospital Frailty Risk Score categories in pneumonia patients

In Kaplan–Meier analysis (Fig. 2), when comparing the categories of the Hospital Frailty Risk Score and Johns Hopkins Claims-Based Frailty, the long-term mortality (primary outcome) rate was 21.0% vs 17.2% in the low-risk group, 43.9% vs 34.9% in the intermediate-risk group, and 65.6% vs 54.3% in the high-risk group, respectively, for AMI patients (log rank p < 0.001 for comparison between categories). In HF patients, when comparing the Hospital Frailty Risk Score and Johns Hopkins Claims-Based Frailty categories, respectively, the long-term mortality (primary outcome) rate was 41.1% vs 35.8% in the low-risk group, 63.9% vs 52.6% in the intermediate-risk group, and 74.7% vs 65.7% in the high-risk group (log rank p < 0.001 for comparison between categories). In pneumonia patients, Hospital Frailty Risk Score and Johns Hopkins Claims-Based Frailty categories, respectively, the long-term mortality (primary outcome) rate was 32.5% and 29.2% in the low-risk group, 60.3% and 49.8% in the intermediate-risk group, and 74.3% and 64.9% in the high-risk group (log rank p < 0.001 for comparison between categories). As shown in eFigure 1, the results of sensitivity analyses were consistent with these findings (all log rank p < 0.001 for comparison between categories).

Observed 1-year readmission and observed 1-year mortality rates for all prespecified subgroups were consistently and significantly associated with increased Hospital Frailty Risk Score and Johns Hopkins Claims-Based Frailty categories for all 3 conditions studied (Table 2).

**Table 2:** Outcomes of the Study Population according to Johns Hopkins Claims-Based Frailty Index and Hospital Frailty Risk Score categories

	Johns Hopkins Claims-Based Frailty Index			
	Low-Risk (<0.12)	Intermediate-Risk (0.12-0.20)	High-Risk (>0.20)	p value
**Acute myocardial infarction**	**n=24,509**	**n=6,667**	**n=3,920**	
Observed 1-year Readmission	11,078 (45.2%)	3,437 (51.6%)	2,361 (60.2%)	<0.001
Observed 1-year Mortality	3,067 (12.5%)	1,672 (25.1%)	1,416 (36.1%)	<0.001
**Heart Failure**	**n=30,622**	**n=16,895**	**n=14,886**	
Observed 1-year Readmission	16,103 (52.6%)	9,611 (56.9%)	9,668 (64.9%)	<0.001
Observed 1-year Mortality	7,686 (25.1%)	6,224 (36.8%)	6,608 (44.4%)	<0.001
**Pneumonia**	**n=60,619**	**n=24,230**	**n=18,600**	
Observed 1-year Readmission	28,172 (46.5%)	12,545 (51.8%)	11,381 (61.2%)	<0.001
Observed 1-year Mortality	12,782 (21.1%)	8,715 (36.0%)	8,522 (45.8%)	<0.001
	**Hospital Frailty Risk Score**			
	**Low-Risk (<5)**	**Intermediate-Risk (5-15)**	**High-Risk (>15)**	
**Acute myocardial infarction**	**n=30,083**	**n=4,561**	**n=452**	
Observed 1-year Readmission	13,119 (43.6%)	3,375 (74.0%)	382 (84.5%)	<0.001
Observed 1-year Mortality	4,836 (16.1%)	1,186 (26.0%)	133 (29.4%)	<0.001
**Heart Failure**	**n=45,995**	**n=14,513**	**n=1,895**	
Observed 1-year Readmission	23,441 (51.0%)	10,408 (71.7%)	1,533 (80.9%)	<0.001
Observed 1-year Mortality	13,871 (30.2%)	5,851 (40.3%)	796 (42.0%)	<0.001
**Pneumonia**	**n=75,498**	**n=24,499**	**n=3,452**	
Observed 1-year Readmission	33,695 (44.6%)	15,696 (64.1%)	2,707 (78.4%)	<0.001
Observed 1-year Mortality	18,160 (24.1%)	10,306 (42.1%)	1,553 (45.0%)	<0.001

After adjustment for age, sex, and covariates, multivariable logistic and Cox regression analyses revealed that higher Hospital Frailty Risk Score and Johns Hopkins Claims-Based Frailty categories were significantly associated with a higher risk of observed 1-year readmission, 1-year mortality, and long-term mortality in the AMI, HF, and pneumonia populations (Table 3).

**Table 3:** Multivariable Logistic and Cox Regression Analyses Results according to Johns Hopkins Claims-Based Frailty Index and Hospital Frailty Risk Score categories

	Johns Hopkins Claims-based Frailty Index					
	Acute Myocardial Infarction (Odds Ratio 95% CI)	p value	Heart Failure (Odds Ratio 95% CI)	p value	Pneumonia (Odds Ratio 95% CI)	p value
**Observed 1-year Readmission**						
Low-Risk (<0.12)	1 (reference)	<0.001	1 (reference)	<0.001	1 (reference)	<0.001
Intermediate-Risk (0.12-0.20)	2.119 (1.945-2.309)		3.031 (2.854-3.219)		2.875 (2.757-2.999)	
High-Risk (>0.20)	3.977 (3.516-4.499)		7.972 (7.320-8.681)		7.243 (6.833-7.677)	
**Observed 1-year Mortality**						
Low-Risk (<0.12)	1 (reference)	<0.001	1 (reference)	<0.001	1 (reference)	<0.001
Intermediate-Risk (0.12-0.20)	2.515 (2.359-2.678)		1.807 (1.733-1.885)		2.390 (2.316-2.467)	
High-Risk (>0.20)	4.552 (4.231-4.797)		2.666 (2.551-2.785)		4.014 (3.879-4.154)	
**Long-Term Mortality (Hazard Ratio 95% CI)**						
Low-Risk (<0.12)	1 (reference)	<0.001	1 (reference)	<0.001	1 (reference)	<0.001
Intermediate-Risk (0.12-0.20)	2.365 (2.253-2.483)		1.703 (1.655-1.753)		2.112 (2.063-2.163)	
High-Risk (>0.20)	4.142 (3.934-4.360)		2.450 (2.380-2.521)		3.347 (3.266-3.430)	
	**Hospital Frailty Risk Score**					
**Observed 1-year Readmission**						
Low-Risk (<5)	1 (reference)	<0.001	1 (reference)	<0.001	1 (reference)	<0.001
Intermediate-Risk (5-15)	3.162 (2.940-3.402)		2.041 (1.951-2.134)		1.827 (1.773-1.883)	
High-Risk (>15)	5.188 (4.032-6.676)		3.199 (2.825-3.624)		3.534 (3.265-3.825)	
**Observed 1-year Mortality**						
Low-Risk (<5)	1 (reference)	<0.001	1 (reference)	<0.001	1 (reference)	<0.001
Intermediate-Risk (5-15)	1.701 (1.582-1.828)		1.711 (1.641-1.785)		2.289 (2.220-2.361)	
High-Risk (>15)	1.819 (1.496-2.111)		1.808 (1.636-1.998)		2.611 (2.438-2.797)	
**Long-Term Mortality (Hazard Ratio 95% CI)**						
Low-Risk (<5)	1 (reference)	<0.001	1 (reference)	<0.001	1 (reference)	<0.001
Intermediate-Risk (5-15)	2.209 (2.104-2.320)		1.929 (1.879-1.981)		2.410 (2.359-2.463)	
High-Risk (>15)	3.275 (2.930-3.660)		2.390 (2.260-2.528)		3.204 (3.071-3.342)	

Models adjusted for age, sex, and covariates

C-statistics for 1-year readmission using only the Hospital Frailty Risk Score (AMI=0.68, HF=0.65, pneumonia=0.65) were better than using only Johns Hopkins Claims-Based Frailty Index (AMI=0.58, HF=0.57, pneumonia=0.65) (DeLong p<0.001). However, for 1-year mortality Johns Hopkins Claims-Based Frailty Index (for 1-year mortality: AMI=0.67, HF=0.62, pneumonia=0.65) (for long-term mortality: AMI=0.68, HF=0.61, pneumonia=0.64) c-statistics were better compared to Hospital Frailty Risk Score (for 1-year mortality: AMI=0.58, HF=0.58, pneumonia=0.63) (for long-term mortality: AMI=0.62, HF=0.58, pneumonia=0.62) (DeLong p<0.001 in all) (Table 4).

**Table 4:** Discrimination and comparison of the Johns Hopkins Claims-Based Frailty Index and Hospital Frailty Risk Score models

	Johns Hopkins Claims-based Frailty Index (C Statistic)	Hospital Frailty Risk Score (C Statistic)	DeLongp-value
**Observed 1-year Readmission**			
Acute Myocardial Infarction	0.58 (0.57-0.59)	0.68 (0.68-0.69)	<0.001
Heart Failure	0.57 (0.57-0.57)	0.66 (0.66-0.67)	<0.001
Pneumonia	0.58 (0.58-0.59)	0.65 (0.65-0.66)	<0.001
**Observed 1-year Mortality**			
Acute Myocardial Infarction	0.67 (0.66-0.67)	0.58 (0.58-0.59)	<0.001
Heart Failure	0.62 (0.61-0.62)	0.58 (0.57-0.58)	<0.001
Pneumonia	0.65 (0.64-0.65)	0.63 (0.62-0.63)	<0.001
**Long-term mortality**			
Acute Myocardial Infarction	0.68 (0.67-0.69)	0.62 (0.61-0.63)	<0.001
Heart Failure	0.61 (0.61-0.62)	0.58 (0.57-0.59)	<0.001
Pneumonia	0.64 (0.63-0.65)	0.62 (0.62-0.63)	<0.001

## 4 Discussion

Prediction of clinical outcomes such as rates of readmission and mortality is crucial for improving healthcare and developing more rational health policies. In our previous study, we showed that the evaluation of clinical 30-day outcomes could be incorrect when traditional risk factors were taken into account, and that frailty provides a more convenient reference for predicting risk adjusted clinical outcomes [14]. However, which frailty calculation method should be used to predict specific clinical outcomes is a controversial issue. In this nationwide study demonstrates that administrative ICD-10-CM codes can be used to evaluate frailty. We observed that two ICD-10-CM based frailty indices (Hospital Frailty Risk Score and Johns Hopkins Claims-Based Frailty Index) varied in terms of discriminatory performance for 1-year readmission (c-statistics ranging 0.57 - 0.68), 1-year mortality (c-statistics ranging 0.58 - 0.67) and long-term mortality (c-statistics ranging 0.58 - 0.68).

Our findings indicate that readmission and mortality rates after AMI, HF, and pneumonia gradually increases with increasing frailty score. We have found that frailty was associated with a 1.5- to 3-fold higher risk of 1-year mortality which is similar with after cardiac surgery patients [25]. Despite a modest agreement between the Hospital Frailty Risk Score and the Johns Hopkins Claims-Based Frailty Index in the target populations, the Hospital Frailty Risk Score had a better discrimination for predicting readmissions, and Johns Hopkins Claims-Based Frailty Index had a better discrimination for predicting mortality. Our findings that the Hospital Frailty Risk Score is a useful predictor of adverse outcomes particularly in readmission were similar with some studies [14,19] but not with others [26,27]. We believe that these findings should be taken into account for a better evaluations of hospital performance.

In our study, the Hospital Frailty Risk Score and the Johns Hopkins Claims-Based Frailty Index was found to have a modest correlation. It has been previously reported that the Hospital Frailty Risk Score showed fair to moderate overlap with ratings based on two other frailty scales as well [19]. In one of the biggest prospective studies for assessment of frailty, Afilalo et al. showed the rate of frailty within the same population ranged between 35% and 74% when evaluated with different prospective indices [5]. This matter of low compatibility between different indices is yet to be clarified. For this study, the ICD-9-CM codes used in the Johns Hopkins Claims-based Frailty Index was converted to corresponding ICD-10-CM codes through an automated process. Previous studies that involved a similar approach to conversion of indices have reported stable transitions with comments on the plurality of ICD-10-CM codes and related challenges [28], [29], [30]. Further studies are needed for the validation of our approach.

The ICD-10-CM codes provide a common language for reporting and monitoring diseases in many countries around the world. These codes are widely used by clinicians, governments, public health authorities and insurance providers to track disease prevalence. Administrative data can also be used to estimate healthcare expenditures and to guide the development of new practices. Using the administrative data for the assessment of frailty will facilitate the capture of clinical and administrative health data and will lead to the ability to evaluate the delivery of best practices for frail individuals. One of the biggest advantage of claims-based frailty scores is that of frailty is that it can be measured using routine data for all patients without a need to utilize manual measurements. This makes claims-based frailty indices immune to disadvantages of alternative indices such as inter-operator reliability and difficulty of implementation. Claims-based data allows for a comprehensive, retrospective evaluation of not only a cross-sectional time period, but a broader time scale. In that manner, a dynamic evaluation can be made by determining the changes in frailty score during patients’ follow-up. Besides, the scope of frailty evaluation in related populations can be further expanded by merging claims-based data with registries and prospective data.

The aging trend links to several factors, particularly to better health care that prolongs life in the country, where longevity has shown a rise in recent years. Currently, Monaco (33.5%), Japan (28.5%) and Germany (22.9%) have the highest proportion of the older adults. Turkish healthcare system has been witnessing great transformation since 2003 with the main purpose of achieving better healthcare outcomes and wider access to health services by the gradual introduction of universal health insurance through organizing, providing, financing for, and delivering health services in an effective, productive, and equitable way under the Health Transformation Program. Turkey's older population, aged 65 years and over, reached to almost 8 million (~9%). The proportion of the elderly population in Turkey is expected to rise to more than 10% in 2025, more than 15% in 2040, more than 20% in 2060 [31]. In generally, the elderly people in rural areas continue their traditional lifestyle by living in packed families, unlike the elderly living in urban areas. An investigation carried out by the State Planning Organization indicates that seventy percent of elderly people live in the same building, street or neighbourhood with their children in Turkey. In addition, because of combinations of cultural and religious practices, elderly people have always been respected and assisted in all periods of Turkish history [32]. Thus, it is very important to define more vulnerable patients, particularly frail ones to support them and provide a prolonged life.

### 4.1 Limitations

This study has several limitations. The data were pulled retrospectively from the nationwide health database (e-Pulse). Since these records were not primarily collected for frailty assessment, the ICD-10-CM codes of study population might be missing or inaccurate to a certain extent. In particular, the principal discharge diagnosis of patients who are hospitalized for more than one reason may be inappropriate. It should be also kept in mind that frailty indices based solely on ICD-10 codes are prone to measurement errors arising from regional variations in coding of diagnoses. We were not able to include other outcomes rather than mortality and readmissions. Therefore, our findings may not be generalizable for other outcomes. Lastly, our analysis was limited to Turkey and may not be applicable to other countries.

## 5 Conclusions

Among Turkish nationwide cohort, claims-based frailty was strongly associated with 1-year readmissions and mortality, and long-term mortality among patients hospitalized for AMI, HF, or pneumonia. The Hospital Frailty Risk Score had a better discrimination for predicting readmissions, and Johns Hopkins Claims-Based Frailty Index had a better discrimination for predicting mortality. Our findings should be taken into account for a better evaluation of hospital performance.

## Author statements (Contributors)

Dr. Kundi had full access to all of the data in the study and takes responsibility for the integrity of the data and the accuracy of the data analysis.

*Concept and design:* Kundi, Coskun, Yesiltepe.

*Acquisition, analysis, or interpretation of data:* Kundi, Coskun, Yesiltepe.

*Drafting of the manuscript:* Kundi, Coskun, Yesiltepe*.*

*Statistical analysis:* Kundi.

*Obtained funding:* Kundi, Yesiltepe.

*Administrative, technical, or material support:* Kundi, Coskun, Yesiltepe.

*Supervision:* Kundi.

*Revision: Kundi, Coskun, Yesiltepe.*

## Funding/Support

This study was supported by funding from The Scientific and Technological Research Council of Turkey (grant 120S422, HK). The data was provided by Republic of Turkey Ministry of Health.

## Ethical approval

Ethical approval for this study was obtained from Ankara City Hospital of ethics committee.

## Data availability statement

Restricted by our data use agreement with the Republic of Turkey Ministry of Health. The data used for this study cannot be made publicly available.

## Declaration of Interests

HK and MY report a grant from The Scientific and Technological Research Council of Turkey (grant 120S422, HK). There are no further disclosures or conflict of interest to report.

## Appendix Supplementary materials

Image, application 1

## Footnotes

Supplementary material associated with this article can be found, in the online version, at doi:10.1016/j.lanepe.2021.100183.
