# Supplementary material for: Association of entirely claims-based frailty indices with long-term outcomes in patients with acute myocardial infarction, heart failure, or pneumonia: a nationwide cohort study in Turkey
Source: Lancet Reg Health Eur. 2021 Jul 29;10:100183. doi: 10.1016/j.lanepe.2021.100183 (PMC8589716; doi:10.1016/j.lanepe.2021.100183)

**Supplemental Files**

**eTable 1.** Codes to identify hospitalizations

|  | **ICD-10-CM Codes** |
| --- | --- |
| **Acute Myocardial Infarction** | I2101 I2102 I2109 I2111 I2119 I2121 I2129 I213 I214 I219 I21A1 I21A9 |
| **Heart Failure** | I110 I130 I132 I501 I509 I5020 I5021 I5022 I5023 I5030 I5031 I5032 I5033 I5040 I5041 I5042 I5043 |
| **Pneumonia** | J120 J121 J122 J123 J1281 J1289 J129 J13 J14 J150 J151 J1520 J15211 J15212 J1529 J153 J154 J155 J156 J157 J158 J159 J160J 168 J180 J181 J182 J188 J189 |

**eTable 2.** List of ICD-10-CM codes to identify covariates

|  | **ICD-10-CM Codes** |
| --- | --- |
| **History of Acute Myocardial Infarction** | I20, I21, I22, I23, I24, I25 |
| **History of Coronary Artery Bypass Graft** | Z951 |
| **History of Valvular Heart Disease** | A520, I091, I098, Q230, Q231, Q232, Q233, Z952, Z953, Z954, I05, I06, I07, I08, I34, I35, I36, I37, I38, I39 |
| **Hypertension** | I11, I12, I13, I15 |
| **Peripheral Vascular Disease** | I731, I738, I739, I771, I790, I792, K551, K558, K559, I70, I71, Z958, Z959 |
| **Cerebrovascular Disease** | H340, I64, I65, I66, I67, I68, I69, G45, G46, I60, I61, I62, I63 |
| **Chronic Obstructive Pulmonary Disease** | I278, I279, J684, J701, J703, J40, J41, J42, J43, J44, J45, J46, J47, J60, J61, J62, J63, J64, J65, J66, J67 |
| **Diabetes Mellitus** | E110, E111, E116, E118, E119, E120, E121, E126, E128, E100, E101, E106, E108, E109, E129, E130, E131, E136, E138, E139, E140, E141, E146, E148, E149, E143, E144, E145, E147, E127, E132, E133, E134, E135, E137, E142, E113, E114, E115, E117, E122, E123, E124, E125, E102, E103, E104, E105, E107, E112 |
| **Obesity** | E66 |
| **Liver Disease** | B18, K73, K74, K700, K701, K702, K703, K709, K713, K714, K715, K717, K760, K762, K763, K764, K768, K769, Z944, I850, I859, I864, I982, K704, K711, K721, K729, K765, K766, K767 |
| **Renal Failure** | N18, N19, N052, N053, N054, N055, N056, N057, N250, I120, I131, N032, N033, N034, N035, N036, N037, Z490, Z491, Z492, Z940, Z992 |
| **Deficiency Anemia** | D50 |
| **Rheumatoid Disease** | M05, M32, M33, M34, M06, M315, M351, M353, M360 |
| **Peptic Ulcer Disease** | K25, K26, K27, K28 |
| **Dementia** |  |
| **Depression** | F32, F33, F204, F313, F314, F315, F341, F412, F432 |
| **Cancer** | C00, C01, C02, C03, C04, C05, C06, C07, C08, C09, C10, C11, C12, C13, C14, C15, C16, C17, C18, C19, C20, C21, C22, C23, C24, C25, C26, C30, C31, C32, C33, C34, C37, C38, C39, C40, C41, C43, C45, C46, C47, C48, C49, C50, C51, C52, C53, C54, C55, C56, C57, C58, C60, C61, C62, C63, C64, C65, C66, C67, C68, C69, C70, C71, C72, C73, C74, C75, C76, C77, C78, C79, C80, C81, C82, C83, C84, C85, C88, C90, C91, C92, C93, C94, C95, C96, C97 |
| **Substance Abuse** | G621, I426, K292, K700, K703, K709, Z502, Z714, Z721, F10, E52, T51, F11, F12, F13, F14, F15, F16, F18, F19, Z715, Z722 |
| **Acquired Immunodeficiency Syndrome** | B20, B21, B22, B24 |

**eTable 3.** List of ICD-10-CM codes, number of points for each to create the Hospital Frailty Risk Score

| **Codes** | **Definition** | **Point** | **Acute Myocardial Infarction n=35,096** | **Heart Failure**  **n=62,403** | **Pneumonia**  **n=103,449** |
| --- | --- | --- | --- | --- | --- |
| **F00** | Dementia in Alzheimer | 7·1 | 545 (1.6%) | 2,914 (4.7%) | 6,713 (6.5%) |
| **G81** | Hemiplegia | 4·4 | 306 (0.9%) | 770 (1.2%) | 1,240 (1.2%) |
| **G30** | Alzheimer's disease | 4·0 | 245 (0.7%) | 914 (1.5%) | 1,618 (1.6%) |
| **I69** | Sequelae of cerebrovascular disease (secondary codes) | 3·7 | 13 (0.0%) | 47 (0.1%) | 66 (0.1%) |
| **R29** | Other symptoms and signs involving the nervous and musculoskeletal systems (R29·6 Tendency to fall) | 3·6 | 2,999 (8.5%) | 7,069 (11.3%) | 12,652 (12.2%) |
| **N39** | Other disorders of urinary system (includes urinary tract infection and urinary incontinence) | 3·2 | 236 (0.7%) | 850 (1.4%) | 1,535 (1.5%) |
| **F05** | Delirium, not induced by alcohol and other psychoactive substances | 3·2 | 345 (1.0%) | 773 (1.2%) | 1,147 (1.1%) |
| **W19** | Unspecified fall | 3·2 | 56 (0.2%) | 55 (0.1%) | 85 (0.1%) |
| **S00** | Superficial injury of head | 3·2 | 295 (0.8%) | 651 (1.0%) | 858 (0.8%) |
| **R31** | Unspecified hematuria | 3·0 | 63 (0.2%) | 367 (0.6%) | 648 (0.6%) |
| **B96** | Other bacterial agents as the cause of diseases classified to other chapters (secondary code) | 2·9 | 11 (0.0%) | 15 (0.0%) | 35 (0.0%) |
| **R41** | Other symptoms and signs involving cognitive functions and awareness | 2·7 | 91 (0.3%) | 364 (0.6%) | 496 (0.5%) |
| **R26** | Abnormalities of gait and mobility | 2·6 | 2,839 (8.1%) | 6,531 (10.5%) | 9,510 (9.2%) |
| **I67** | Other cerebrovascular diseases | 2·6 | 15 (0.0%) | 24 (0.0%) | 42 (0.0%) |
| **R56** | Convulsions, not elsewhere classified | 2·6 | 172 (0.5%) | 430 (0.7%) | 854 (0.8%) |
| **R40** | Somnolence, stupor and coma | 2·5 | 2 (0.0%) | 1 (0.0%) | 1 (0.0%) |
| **T83** | Complications of genitourinary prosthetic devices, implants and grafts | 2·4 | 85 (0.2%) | 126 (0.2%) | 220 (0.2%) |
| **S06** | Intracranial injury | 2·4 | 47 (0.1%) | 124 (0.2%) | 227 (0.2%) |
| **S42** | Fracture of shoulder and upper arm | 2·3 | 1,363 (3.9%) | 4,834 (7.7%) | 6,217 (6.0%) |
| **E87** | Other disorders of fluid, electrolyte and acid-base balance | 2·3 | 449 (1.3%) | 1,110 (1.8%) | 1,742 (1.7%) |
| **M25** | Other joint disorders, not elsewhere classified | 2·3 | 9 (0.0%) | 41 (0.1%) | 44 (0.0%) |
| **E86** | Volume depletion | 2·3 | 221 (0.6%) | 1,006 (1.6%) | 1,621 (1.6%) |
| **R54** | Senility | 2·2 | 90 (0.3%) | 468 (0.7%) | 1,061 (1.0%) |
| **F03** | Unspecified dementia | 2·1 | 20 (0.1%) | 76 (0.1%) | 126 (0.1%) |
| **W18** | Other fall on same level | 2·1 | 24 (0.1%) | 14 (0.0%) | 14 (0.0%) |
| **Z75** | Problems related to medical facilities and other health care | 2·0 | 30 (0.1%) | 141 (0.2%) | 182 (0.2%) |
| **F01** | Vascular dementia | 2·0 | 8 (0.0%) | 7 (0.0%) | 21 (0.0%) |
| **S80** | Superficial injury of lower leg | 2·0 | 226 (0.6%) | 1,257 (2.0%) | 1,146 (1.1%) |
| **L03** | Cellulitis | 2·0 | 5 (0.0%) | 18 (0.0%) | 24 (0.0%) |
| **H54** | Blindness and low vision | 1·9 | 80 (0.2%) | 334 (0.5%) | 548 (0.5%) |
| **E53** | Deficiency of other B group vitamins | 1·9 | 245 (0.7%) | 914 (1.5%) | 1,618 (1.6%) |
| **G20** | Parkinson's disease | 1·8 | 201 (0.6%) | 875 (1.4%) | 1,862 (1.8%) |
| **R55** | Syncope and collapse | 1·8 | 840 (2.4%) | 1,141 (1.8%) | 1,017 (1.0%) |
| **S22** | Fracture of rib(s), sternum and thoracic spine | 1·8 | 78 (0.2%) | 167 (0.3%) | 382 (0.4%) |
| **K59** | Other functional intestinal disorders | 1·8 | 888 (2.5%) | 3,047 (4.9%) | 4,665 (4.5%) |
| **N17** | Acute renal failure | 1·8 | 2,381 (6.8%) | 9,185 (14.7%) | 10,911 (10.5%) |
| **L89** | Decubitus ulcer | 1·7 | 240 (0.7%) | 1,406 (2.3%) | 2,810 (2.7%) |
| **Z22** | Carrier of infectious disease | 1·7 | 2 (0.0%) | 19 (0.0%) | 26 (0.0%) |
| **B95** | Streptococcus and staphylococcus as the cause of diseases classified to other chapters | 1·7 | 13 (0.0%) | 78 (0.1%) | 109 (0.1%) |
| **L97** | Ulcer of lower limb, not elsewhere classified | 1.6 | 2 (0.0%) | 28 (0.0%) | 21 (0.0%) |
| **R44** | Other symptoms and signs involving general sensations and perceptions | 1·6 | 115 (0.3%) | 431 (0.7%) | 877 (0.8%) |
| **K26** | Duodenal ulcer | 1·6 | 31 (0.1%) | 74 (0.1%) | 101 (0.1%) |
| **I95** | Hypotension | 1·6 | 540 (1.5%) | 1,439 (2.3%) | 1,771 (1.7%) |
| **N19** | Unspecified renal failure | 1·6 | 471 (1.3%) | 1,828 (2.9%) | 1,735 (1.7%) |
| **A41** | Other septicemia | 1·6 | 955 (2.7%) | 3,821 (6.1%) | 7,150 (6.9%) |
| **X59** | Exposure unspecified factor | 1·5 | 0 (0.0%) | 4 (0.0%) | 15 (0.0%) |
| **Z87** | Personal history of other diseases and conditions | 1·5 | 3,232 (9.2%) | 12,839 (20.6%) | 20,165 (19.5%) |
| **J96** | Respiratory failure, not elsewhere classified | 1·5 | 43 (0.1%) | 380 (0.6%) | 258 (0.2%) |
| **M19** | Other arthrosis | 1·5 | 285 (0.8%) | 936 (1.5%) | 1,809 (1.7%) |
| **G40** | Epilepsy | 1·5 | 219 (0.6%) | 721 (1.2%) | 1,401 (1.4%) |
| **M81** | Osteoporosis without pathological fracture | 1·4 | 390 (1.1%) | 1,270 (2.0%) | 1,735 (1.7%) |
| **S72** | Fracture of femur | 1·4 | 68 (0.2%) | 198 (0.3%) | 302 (0.3%) |
| **S32** | Fracture of lumbar spine and pelvis | 1·4 | 140 (0.4%) | 403 (0.6%) | 421 (0.4%) |
| **E16** | Other disorders of pancreatic internal secretion | 1·4 | 955 (2.7%) | 3,821 (6.1%) | 7,150 (6.9%) |
| **R94** | Abnormal results of function studies | 1·4 | 506 (1.4%) | 1,231 (2.0%) | 1,620 (1.6%) |
| **N18** | Chronic renal failure | 1·4 | 1,693 (4.8%) | 7,413 (11.9%) | 6,503 (6.3%) |
| **R33** | Retention of urine | 1·3 | 84 (0.2%) | 145 (0.2%) | 298 (0.3%) |
| **R69** | Unknown and unspecified causes of morbidity | 1·3 | 4 (0.0%) | 3 (0.0%) | 23 (0.0%) |
| **N28** | Other disorders of kidney and ureter, not elsewhere classified | 1·3 | 197 (0.6%) | 456 (0.7%) | 474 (0.5%) |
| **G31** | Other degenerative diseases of nervous system, not elsewhere classified | 1·2 | 7 (0.0%) | 33 (0.1%) | 63 (0.1%) |
| **R32** | Unspecified urinary incontinence | 1·2 | 185 (0.5%) | 975 (1.6%) | 1,547 (1.5%) |
| **S09** | Other and unspecified injuries of head | 1·2 | 1 (0.0%) | 13 (0.0%) | 36 (0.0%) |
| **R45** | Symptoms and signs involving emotional state | 1·2 | 65 (0.2%) | 162 (0.3%) | 205 (0.2%) |
| **G45** | Transient cerebral ischemic attacks and related syndromes | 1·2 | 413 (1.2%) | 596 (1.0%) | 626 (0.6%) |
| **Y95** | Nosocomial condition | 1·2 | 0 (0.0%) | 0 (0.0%) | 3 (0.0%) |
| **Z60** | Problems related to social environment | 1·1 | 1 (0.0%) | 1 (0.0%) | 3 (0.0%) |
| **Z74** | Problems related to care-provider dependency | 1·1 | 13 (0.0%) | 104 (0.2%) | 224 (0.2%) |
| **M79** | Other soft tissue disorders, not elsewhere classified | 1·1 | 3,207 (9.1%) | 5,532 (8.9%) | 8,600 (8.3%) |
| **W06** | Fall involving bed | 1.1 | 4 (0.0%) | 15 (0.0%) | 12 (0.0%) |
| **S01** | Open wound of head | 1.1 | 18 (0.1%) | 24 (0.0%) | 42 (0.0%) |
| **A09** | Diarrhea and gastroenteritis of presumed infectious origin | 1.1 | 622 (1.8%) | 630 (1.0%) | 1,118 (1.1%) |
| **J18** | Pneumonia, organism unspecified | 1·1 | 5,467 (15.6%) | 19,992 (32.0%) | 86,628 (83.7%) |
| **A04** | Other bacterial intestinal infections | 1·1 | 131 (0.4%) | 135 (0.2%) | 316 (0.3%) |
| **R02** | Gangrene not classified anywhere | 1·0 |  |  |  |
| **J69** | Pneumonitis due to solids and liquids | 1·0 | 30 (0.1%) | 181 (0.3%) | 475 (0.5%) |
| **R47** | Speech disturbances, not elsewhere classified | 1·0 | 68 (0.2%) | 136 (0.2%) | 182 (0.2%) |
| **E55** | Vitamin D deficiency | 1·0 | 1,069 (3.0%) | 3,517 (5.6%) | 5,607 (5.4%) |
| **Z93** | Artificial opening status | 1·0 | 189 (0.5%) | 800 (1.3%) | 2,235 (2.2%) |
| **R63** | Symptoms and signs concerning food and fluid intake | 0·9 | 61 (0.2%) | 259 (0.4%) | 481 (0.5%) |
| **H91** | Other hearing loss | 0·9 | 33 (0.1%) | 108 (0.2%) | 209 (0.2%) |
| **W10** | Fall on and from stairs and steps | 0·9 | 12 (0.0%) | 9 (0.0%) | 11 (0.0%) |
| **W01** | Fall on same level from slipping, tripping and stumbling | 0·9 | 37 (0.1%) | 99 (0.2%) | 94 (0.1%) |
| **E05** | Thyrotoxicosis [hyperthyroidism] | 0·9 | 200 (0.6%) | 877 (1.4%) | 1,076 (1.0%) |
| **M41** | Scoliosis | 0·9 | 24 (0.1%) | 41 (0.1%) | 80 (0.1%) |
| **R13** | Dysphagia | 0·8 | 34 (0.1%) | 108 (0.2%) | 296 (0.3%) |
| **Z99** | Dependence on enabling machines and devices | 0·8 | 29 (0.1%) | 94 (0.2%) | 196 (0.2%) |
| **M80** | Osteoporosis with pathological fracture | 0·8 | 16 (0.0%) | 92 (0.1%) | 145 (0.1%) |
| **K92** | Other diseases of digestive system | 0·8 | 616 (1.8%) | 1,907 (3.1%) | 1,975 (1.9%) |
| **I63** | Cerebral Infarction | 0·8 | 478 (1.4%) | 1,272 (2.0%) | 1,667 (1.6%) |
| **N20** | Calculus of kidney and ureter | 0·7 | 215 (0.6%) | 338 (0.5%) | 627 (0.6%) |
| **F10** | Mental and behavioral disorders due to use of alcohol | 0·7 | 2 (0.0%) | 7 (0.0%) | 15 (0.0%) |
| **Y84** | Other medical procedures as the cause of abnormal reaction of the patient | 0·7 | 2 (0.0%) | 12 (0.0%) | 21 (0.0%) |
| **R00** | Abnormalities of heart beat | 0·7 | 3,959 (11.3%) | 5,362 (8.6%) | 3,721 (3.6%) |
| **J22** | Unspecified acute lower respiratory infection | 0·7 | 692 (2.0%) | 1,849 (3.0%) | 3,470 (3.4%) |
| **R79** | Other abnormal findings of blood chemistry | 0·6 | 6 (0.0%) | 14 (0.0%) | 23 (0.0%) |
| **Z73** | Problems related to life-management difficulty | 0·6 | 0 (0.0%) | 0 (0.0%) | 0 (0.0%) |
| **Z91** | Personal history of risk-factors, not elsewhere classified | 0·5 | 16 (0.0%) | 10 (0.0%) | 3 (0.0%) |
| **S51** | Open wound of forearm | 0·5 | 0 (0.0%) | 2 (0.0%) | 7 (0.0%) |
| **F32** | Depressive episode | 0·5 | 246 (0.7%) | 1,312 (2.1%) | 1,778 (1.7%) |
| **M48** | Spinal stenosis | 0·5 | 87 (0.2%) | 136 (0.2%) | 252 (0.2%) |
| **E83** | Disorders of mineral metabolism | 0·4 | 80 (0.2%) | 266 (0.4%) | 418 (0.4%) |
| **M15** | Polyarthrosis | 0·4 | 16 (0.0%) | 106 (0.2%) | 109 (0.1%) |
| **D64** | Other anemias | 0·4 | 1,882 (5.4%) | 7,121 (11.4%) | 8,454 (8.2%) |
| **L08** | Other local infections of skin and subcutaneous tissue | 0·4 | 242 (0.7%) | 946 (1.5%) | 1,126 (1.1%) |
| **R11** | Nausea and vomiting | 0·3 | 4,738 (13.5%) | 3,383 (5.4%) | 5,755 (5.6%) |
| **K52** | Other noninfective gastroenteritis and colitis | 0·3 | 714 (2.0%) | 1,323 (2.1%) | 2,414 (2.3%) |
| **R50** | Fever of unknown origin | 0·1 | 3,004 (8.6%) | 3,358 (5.4%) | 8,965 (8.7%) |
| **U80** | Agent resistant to penicillin and related antibiotics | 0 | 0 (0.0%) | 0 (0.0%) | 0 (0.0%) |

**eTable4.** List of ICD-9 codes and appropriate ICD-10 codes to create Johns Hopkins Claims Based Frailty Index

|  | **ICD-9-CM** | **ICD-10-CM** |
| --- | --- | --- |
| **Arthritis** | 7140 7141 7142 71430 71431 71432 71433 7144 71481 71489 7149 7200 71500 71504 71509 71510 71511 71512 71513 71514 71515 71516 71517 71518 71520 71521 71522 71523 71524 71525 71526 71527 71528 71530 71531 71532 71533 71534 71535 71536 71537 71538 71580 71589 71590 71591 71592 71593 71594 71595 71596 71597 71598 V134 | M0500 M0510 M0530 M0560 M061 M064 M069 M0800 M083 M0840 M1200 M150 M151 M152 M153 M158 M159 M1610 M167 M169 M1710 M175 M179 M189 M19019 M19029 M19039 M19049 M19079 M19219 M19229 M19239 M19249 M19279 M1990 M1991 M1993 M459 Z8739 |
| **Cognitive Impairment** | 2900 29010 29011 29012 29013 29020 29021 2903 29040 29041 29042 29043 2908 2909 2930 2931 2940 2941 29410 29411 29420 29421 2948 2949 3100 3102 3108 31081 31089 3109 3310 3311 33111 33119 3312 33182 797 | F0390 F0150 F0151 F05 F04 F0280 F0281 F0391 F060 F068 F070 F0781 F482 F0789 F09 G309 G3101 G3109 G311 G3183 R4181 F0390 |
| **Congestive Heart Failure** | 39891 4280 4281 42820 42821 42822 42823 42830 42831 42832 42833 42840 42841 42843 4289 | I0981 I501 I5020 I5021 I5022 I5023 I5030 I5031 I5032 I5033 I5040 I5041 I5042 I5043 I509 |
| **Depression** | 3090 3091 30922 30923 30924 30928 30929 3093 3094 30982 30983 30989 3099 29383 29600 29601 29602 29603 29604 29605 29606 29610 29611 29612 29613 29614 29615 29616 29620 29621 29622 29623 29624 29625 29626 29630 29631 29632 29633 29634 29635 29636 29640 29641 29642 29643 29644 29645 29646 29650 29651 29652 29653 29654 29655 29656 29660 29661 29662 29663 29664 29665 29666 2967 29680 29681 29682 29689 29690 29699 3004 311 | F0630 F3010 F3011 F3012 F3013 F302 F303 F304 F3110 F3111 F3112 F3113 F312 F3130 F3131 F3132 F314 F315 F3160 F3161 F3162 F3163 F3164 F3173 F3174 F3175 F3176 F3177 F3181 F319 F320 F321 F322 F323 F324 F325 F328 F329 F330 F331 F332 F333 F3341 F3342 F339 F341 F348 F39 F4320 F4321 F4322 F4323 F4324 F4325 F4329 F438 F948 |
| **Falls** | E8800 E8801 E8809 E8810 E8811 E882 E8830 E8831 E8832 E8839 E8840 E8841 E8842 E8843 E8844 E8845 E8846 E8849 E885 E8850 E8851 E8852 E8853 E8854 E8859 E8860 E8869 E888 E8880 E8881 E8888 E8889 E9681 E9870 E9871 E9872 E9879 | W100XXA W101XXA W108XXA W11XXXA W12XXXA W139XXA W1692XA W170XXA W171XXA W172XXA W098XXA W15XXXA W07XXXA W050XXA W06XXXA W08XXXA W1811XA W14XXXA W1781XA W1789XA V00141A V00111A V00151A V00131A V00321A V00312A W1849XA W03XXXA V00388A W01110A W01198A W1830XA W19XXXA Y01XXXA Y30XXXA |
| **Impaired Mobility** | V463 7812 V5781 | Z993 B070 Z48810 |
| **Musculoskeletal problems** | 7130 7131 7132 7133 7134 7135 7136 7137 7138 71600 71601 71602 71603 71604 71605 71606 71607 71608 71609 71620 71621 71622 71623 71624 71625 71626 71627 71629 71629 71630 71631 71632 71633 71634 71635 71636 71637 71638 71639 71640 71641 71642 71643 71644 71645 71646 71647 71648 71649 71650 71651 71652 71653 71654 71655 71656 71657 71658 71659 71660 71661 71662 71663 71664 71665 71666 71667 71668 71680 71681 71862 71683 71684 71685 71686 71687 71688 71689 71690 71691 71692 71693 71694 71695 71696 71697 71698 71699 71810 71811 71812 71813 71814 71815 71817 71818 71819 71820 71821 71822 71823 71824 71825 71826 71827 71828 71829 71850 71851 71852 71853 71854 71855 71856 71857 71858 71859 71860 71865 71870 71871 71872 71873 71874 71875 71876 71877 71878 71879 71880 71881 71882 71883 71884 71885 71886 71887 71888 71889 71890 71891 71892 71893 71894 71895 71897 71898 71899 71900 71901 71902 71903 71904 71905 71906 71907 71908 71909 71910 71911 71912 71913 71914 71915 71916 71917 71918 71919 71920 71921 71922 71923 71924 71925 71926 71927 71928 71929 71930 71931 71932 71933 71934 71935 71936 71937 71938 71939 71940 71941 71942 71943 71944 71945 71946 71947 71948 71949 71950 71951 71952 71953 71954 71955 71956 71957 71958 71959 71960 71961 71962 71963 71964 71965 71966 71967 71968 71969 7197 71970 71975 71976 71977 71978 71979 71980 71981 71982 71983 71984 71985 71986 71987 71988 71989 71990 71991 71992 71993 71994 71995 71996 71997 71998 71999 7201 7202 72081 72089 7209 7210 7211 7212 7213 72141 72142 7215 7216 7217 7218 72190 72191 7220 72210 72211 7222 72230 72231 72232 72239 7224 72251 72252 7226 72270 72271 72272 72273 72280 72281 72282 72283 72290 72291 72292 72293 7230 7231 7232 7233 7234 7235 7236 7237 7238 7239 72400 72401 72402 72403 72409 7241 7242 7243 7244 7245 7246 72470 72471 72479 7248 7249 73300 73301 73302 73393 73309 7331 73310 73311 73312 73313 73314 73315 73316 73319 73393 73394 73395 73396 73397 73398 V1351 4350 4351 4352 4353 4358 4359 | G450 G451 G458 G459 I67848 M0200 M0220 M029 M1210 M12119 M12129 M12139 M12149 M12159 M12169 M12179 M1218 M1219 M1220 M12219 M12229 M12239 M12249 M12259 M12269 M12279 M1228 M1229 M1230 M12319 M12329 M12339 M12349 M12359 M12369 M12379 M1238 M1239 M1240 M12419 M12429 M12439 M12449 M12459 M12469 M12479 M1248 M1249 M1280 M12819 M12829 M12839 M12849 M12859 M12869 M12879 M1288 M1289 M129 M130 M1310 M13119 M13129 M13139 M13149 M13159 M13169 M13179 M1380 M13819 M13829 M13839 M13849 M13859 M13869 M13879 M1388 M1389 M1460 M1480 M2350 M238X9 M2400 M24019 M24029 M24039 M24049 M24059 M24073 M24076 M2408 M2430 M24319 M24329 M24339 M24349 M24359 M24369 M24373 M24376 M2460 M24619 M24629 M24639 M24649 M24659 M24669 M24673 M24676 M247 M2480 M24819 M24829 M24839 M24849 M24859 M24873 M24876 M249 M2500 M25019 M25029 M25039 M25049 M25059 M25069 M25073 M25076 M2508 M2510 M25119 M25129 M25139 M25149 M25159 M25169 M25173 M25176 M2518 M2540 M25419 M25429 M25439 M25449 M25459 M25469 M25473 M25476 M2548 M2550 M25519 M25529 M25539 M25559 M25569 M25579 M2560 M25619 M25629 M25639 M25649 M25659 M25669 M25673 M25676 M2580 M25819 M25829 M25839 M25849 M25859 M25869 M25879 M259 M362 M363 M364 M4327 M4328 M436 M438X9 M4600 M461 M4640 M4645 M4647 M4680 M4690 M4710 M4712 M4714 M4716 M47812 M47814 M47817 M47819 M4800 M4802 M4804 M4806 M4808 M4810 M4820 M4830 M4840XA M4841XA M4842XA M4843XA M4844XA M4845XA M4846XA M4847XA M4848XA M4850XA M489 M4980 M5000 M5020 M5030 M5080 M5090 M5104 M5105 M5106 M5107 M5125 M5126 M5127 M5134 M5135 M5136 M5137 M5144 M5145 M5146 M5147 M5184 M5185 M5186 M5187 M519 M530 M531 M532X7 M532X8 M533 M5382 M539 M5402 M5408 M5412 M5413 M5414 M5415 M5416 M5417 M542 M5430 M545 M546 M5489 M549 M6788 M79643 M79646 M8008XA M810 M818 M8430XA M84319A M84329A M84339A M84343A M84350A M84353A M84359A M84369A M84373A M84376A M84379A M8438XA M8440XA M84419A M84429A M84439A M84453A M84459A M84469A M84479A M8448XA M8468XA M961 R262 R294 R29898 Z87311 |
| **Paranoia** | 29381 29382 29500 29501 29502 29503 29504 29505 29510 29511 29512 29513 29514 29515 29520 29521 29522 29523 29524 29525 29530 29531 29532 29533 29534 29535 29540 29541 29542 29543 29544 29545 29550 29551 29552 29553 29554 29555 29560 29561 29562 29563 29564 29565 29570 29571 29572 29573 29574 29575 29580 29581 29582 29583 29584 29585 29590 29591 29592 29593 29594 29595 2970 2971 2972 2973 2978 2979 2980 2981 2982 2983 2984 2988 2989 | F060 F062 F200 F201 F202 F205 F2081 F2089 F209 F22 F23 F24 F259 F28 F29 F323 F333 F4489 |
| **Chronic skin ulcer disease** | 7070 70700 70701 70702 70703 70704 70705 70706 70707 70709 7071 70710 70711 70712 70713 70714 70715 70719 70720 70721 70722 70723 70724 70725 7078 7079 | L8990 L89009 L89119 L89129 L89139 L89149 L89159 L89209 L89309 L89509 L89609 L89819 L89899 L97909 L97109 L97209 L97309 L97409 L97509 L97809 L98419 L98429 L98499 |
| **Stroke** | 34660 34661 34662 34663 430 431 4320 4321 4329 43301 43311 43321 43331 43381 43391 4340 43400 43401 4341 43410 43411 4349 43490 43491 436 438 4380 43810 43811 43812 43813 43814 43819 43820 43821 43822 43840 43841 43842 43850 43851 43852 43853 4386 4387 43881 43882 43883 43884 43885 43889 4389 | G43601 G43609 G43611 G43619 I609 I619 I6200 I621 I629 I63019 I63119 I63219 I63139 I6320 I6322 I63239 I6330 I6340 I6350 I6359 I6609 I669 I6789 I69898 I6990 I6991 I69920 I69921 I69922 I69923 I69928 I69941 I69942 I69943 I69944 I69949 I69951 I69952 I69953 I69954 I69959 I69961 I69962 I69963 I69964 I69965 I69969 I69990 I69991 I69992 I69993 I69998 |
| **Urinary tract infection** | 03284 59000 59001 59010 59011 5902 5903 59080 59081 5909 5950 5951 5952 5953 5954 59581 59582 59589 5959 5970 59780 59781 59789 59800 59801 5990 | A3685 N10 N110 N118 N12 N151 N159 N16 N2884 N2885 N2886 N3000 N3001 N3010 N3011 N3020 N3021 N3030 N3031 N3040 N3041 N3080 N3081 N3090 N3091 N340 N341 N342 N343 N35111 N37 N390 |
| **Parkinson** | 3320 3321 3330 | G20 G2111 G2119 G218 G230 G231 G232 G238 |
| **Skin and Soft tissue infection** | 0201 0210 0220 0311 03285 035 0390 6800 6801 6802 6803 6804 6805 6806 6807 6808 6809 68100 68101 68102 68110 68111 6819 6820 6821 6822 6823 6824 6825 6826 6827 6828 6829 684 6850 6851 6860 68600 68601 68609 6861 6868 6869 | A201 A210 A220 A311 A363 A46 L081 E832 K122 L0100 L0103 L0202 L0203 L0212 L0213 L02221 L02222 L02223 L02224 L02225 L02226 L02229 L02231 L02232 L02233 L02234 L02235 L02236 L02239 L0233 L02429 L02439 L02529 L02539 L02629 L02639 L02821 L02828 L02831 L02838 L0292 L0293 L03019 L03029 L03039 L03049 L03119 L03129 L03211 L03212 L03221 L03222 L03317 L03319 L03329 L03811 L03818 L03891 L03898 L0390 L0391 L0501 L0502 L0591 L0592 L080 L0889 L089 L88 L980 |
| **Pneumonia** | 00322 0203 0204 0205 0212 0221 0310 0391 0521 0551 0730 0830 1124 1140 1144 1145 11505 11515 11595 1304 1363 4800 4801 4802 4803 4808 4809 481 4820 4821 4822 4823 48230 48231 48232 48239 4824 48240 48241 48242 48249 4828 48281 48282 48283 48284 48289 4829 483 4830 4831 4838 4841 4843 4845 4846 4847 4848 485 486 5130 5171 | A0222 A202 A212 A221 A310 A420 B012 B052 A70 A78 B371 B380 B381 B382 B392 B395 J17 B399 B583 B59 J120 J121 J122 J1281 J1289 J129 J13 J181 J150 J151 J14 J154 J153 J1520 J15211 J15212 J1529 J158 J155 J156 A481 J158 J159 J157 J160 J168 B250 A3791 A221 B440 J180 J189 J850 J851 J852 |
| **Gout or other crystal-induced arthropathy** | 2740 27400 27401 27402 27403 27410 27411 27419 27481 27482 27489 2749 71210 71211 71212 71213 71214 71215 71216 71217 71218 71219 71220 71221 71222 71223 71224 71225 71226 71227 71228 71229 71230 71231 71232 71233 71234 71235 71236 71237 71238 71239 71280 71281 71282 71283 71284 71285 71286 71287 71288 71289 71290 71291 71292 71293 71294 71295 71296 71297 71298 71299 | M1000 M1030 M1040 M109 M1120 M11219 M11229 M11239 M11249 M11259 M11269 M11279 M1128 M1129 M1180 M11819 M11829 M11839 M11849 M11859 M11869 M11879 M1188 M1189 M119 M1A00X1 M1A20X1 M1A30X1 M1A40X1 M1A9XX0 M1A9XX1 N200 |
| **Mycoses** | 1100 1101 1102 1103 1104 1105 1106 1108 1109 1110 1111 1112 1113 1118 1119 1120 1121 1122 1123 1125 11282 11284 11285 11289 1129 1141 1143 1149 11500 11509 11510 11519 11590 11599 1160 1161 1162 1170 1171 1172 1173 1174 1175 1176 1177 1178 1179 118 | B350 B351 B352 B356 B353 B355 B358 B359 B360 B361 B362 B363 B368 B369 B370 B3783 B373 B3742 B3749 B372 B377 B3784 B3781 B3782 B3789 B379 B383 B3889 B389 B394 B393 B395 B399 B409 B410 B419 B480 B481 B420 B421 B427 B429 B439 B449 B470 B450 B457 B459 B482 B469 B488 B49 |

**eTable 5.** Johns Hopkins Claims-based Frailty Index algorithm

| **β-coefficient** | **Variable** | **Acute Myocardial Infarction n=35,096** | **Heart Failure**  **n=62,403** | **Pneumonia**  **n=103,449** |
| --- | --- | --- | --- | --- |
| 1.24 | Impaired mobility | 3 (0.0%) | 16 (0.0%) | 35 (0.0%) |
| 0.54 | Depression | 219 (0.6%) | 1,166 (1.9%) | 1,717 (1.7%) |
| 0.50 | Congestive Heart Failure | 2,247 (6.4%) | 12,736 (20.4%) | 6,079 (5.9%) |
| 0.50 | Parkinson’s disease | 203 (0.6%) | 877 (1.4%) | 1,865 (1.8%) |
| -0.49 | White race | 35,096 (100.0%) | 62,403 (100.0%) | 103,449 (100.0%) |
| 0.43 | Arthritis (any type) | 204 (0.6%) | 527 (0.8%) | 1,224 (1.2%) |
| 0.33 | Cognitive impairment | 268 (0.8%) | 1,460 (2.3%) | 3,368 (3.3%) |
| 0.31 | Charlson comorbidity index (>0, 0) | 35,096 (100.0%) | 61,854 (99.1%) | 76,389 (73.8%) |
| 0.28 | Stroke | 3,624 (10.3%) | 8,141 (13.0%) | 11,798 (11.4%) |
| 0.24 | Paranoia | 37 (0.1%) | 217 (0.3%) | 339 (0.3%) |
| 0.23 | Chronic skin ulcer | 252 (0.7%) | 1,462 (2.3%) | 2,857 (2.8%) |
| 0.21 | Pneumonia | 4,212 (12.0%) | 12,425 (19.9%) | 28,972 (28.0%) |
| -0.19 | Male sex | 18,567 (52.9%) | 28,622 (45.9%) | 52,683 (50.9%) |
| 0.18 | Skin and soft tissue infection | 138 (0.4%) | 619 (1.0%) | 728 (0.7%) |
| 0.14 | Mycoses | 205 (0.6%) | 1,213 (1.9%) | 1,799 (1.7%) |
| 0.09 | Age (for every 1 year increase) | 74.9 (7.3) | 77.9 (7.6) | 77.4 (7.8) |
| 0.09 | Admission in past 6 months | 21,803 (62.1%) | 42,163 (67.6%) | 64,242 (62.1%) |
| 0.08 | Gout or other crystal-induced arthropathy | 193 (0.5%) | 827 (1.3%) | 684 (0.7%) |
| 0.08 | Falls | 415 (1.2%) | 975 (1.6%) | 1,410 (1.4%) |
| 0.05 | Musculoskeletal problems | 877 (2.5%) | 1,890 (3.0%) | 2,807 (2.7%) |
| 0.05 | Urinary tract infection | 2,688 7.7%) | 6,077 9.7%) | 11,122 (10.8%) |
| -9 | intercept |  |  |  |
| **score** = -9 + **1.24*** Impaired mobility + **0.54*** Depression + **0.50***Congestive Heart Failure + **0.50***Parkinson’s disease  + (-**0.49**)* White race + **0.43***Arthritis (any type) + **0.33***Cognitive impairment + **0.31***Charlson comorbidity index (>0, 0) + **0.28***Stroke + **0.24***Paranoia + **0.23***Chronic skin ulcer + **0.21*** Pneumonia + (-**0.19**)*Male + **0.18*** Skin and soft tissue infection + **0.14***Mycoses + **0.09***Age + **0.09***Admission in past 6 months + **0.08***Gout or other crystal-induced arthropathy + **0.08***Falls + **0.05*** Musculoskeletal problems + **0.05***Urinary tract infection | | | | |
| **index** = exp(score) / (1+exp(score)) | | | | |

**eFigure 1A.** Kaplan-Meier mortality curves according to Johns Hopkins Claims-Based Frailty and Hospital Frailty Risk Score categories in AMI patients (sensitivity analyses)

**
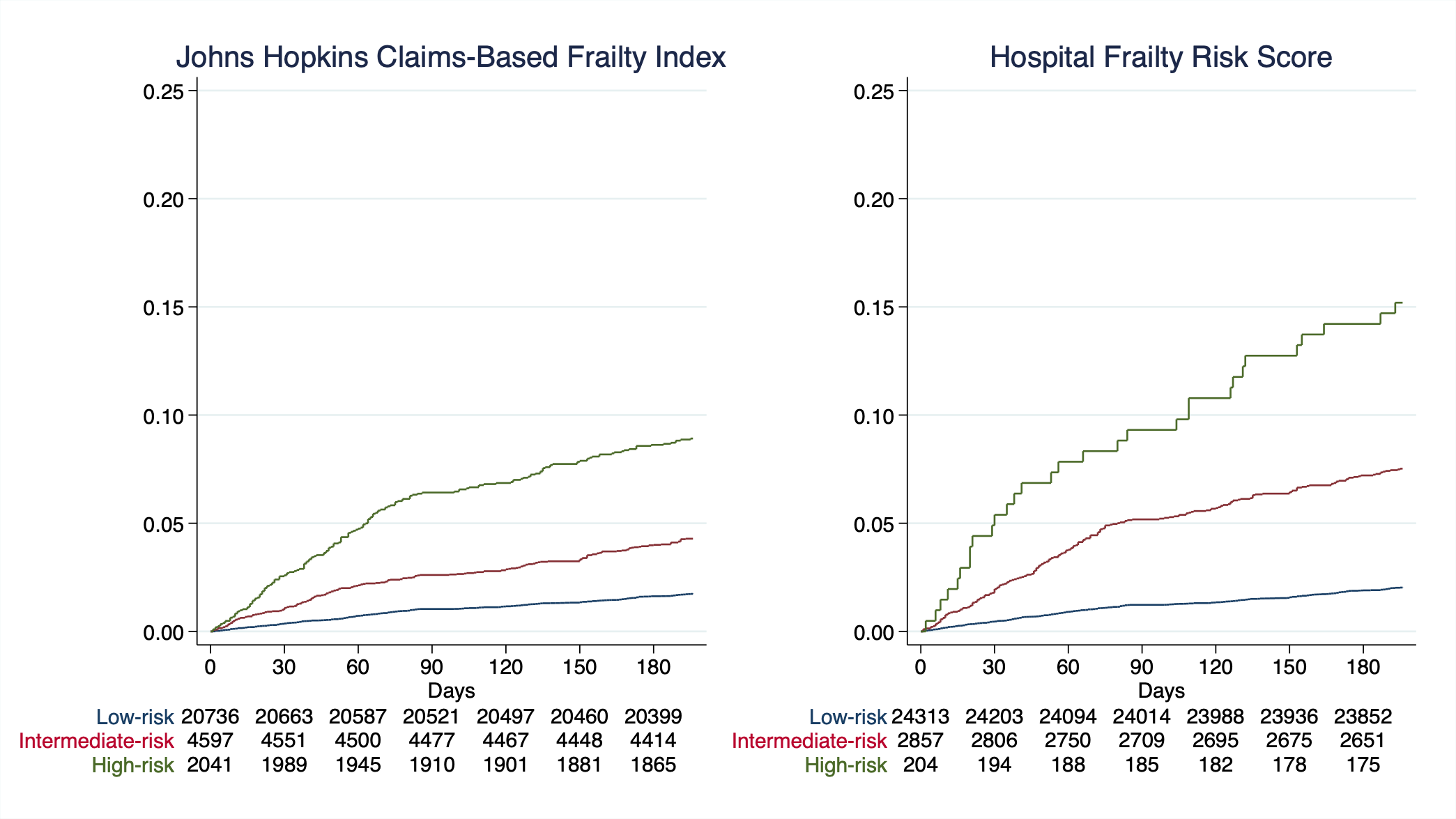
**

**eFigure 1b.** Kaplan-Meier mortality curves according to Johns Hopkins Claims-Based Frailty and Hospital Frailty Risk Score categories in HF patients (sensitivity analyses)

**
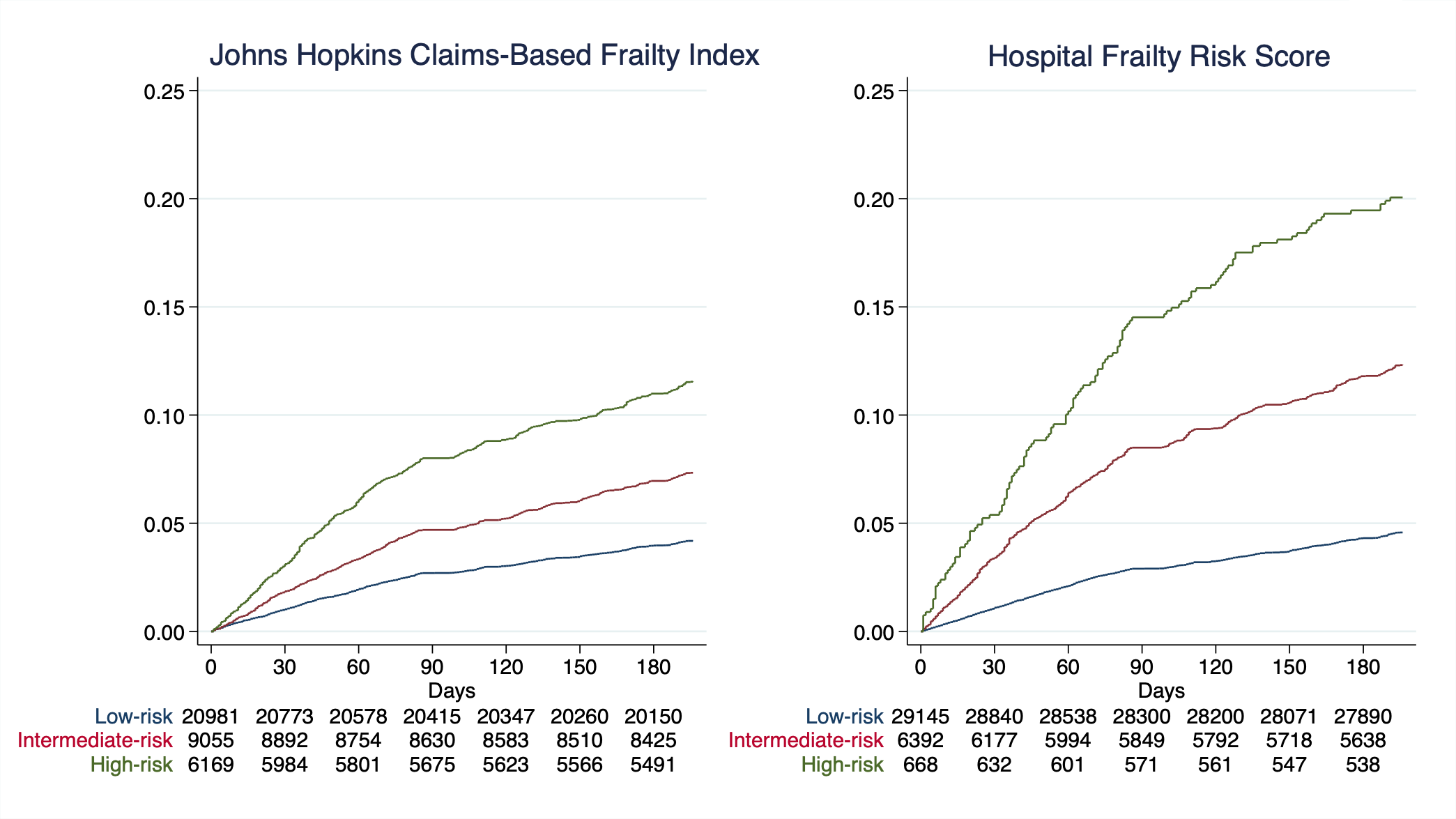
**

**eFigure 1c.** Kaplan-Meier mortality curves according to Johns Hopkins Claims-Based Frailty and Hospital Frailty Risk Score categories in pneumonia patients (sensitivity analyses)


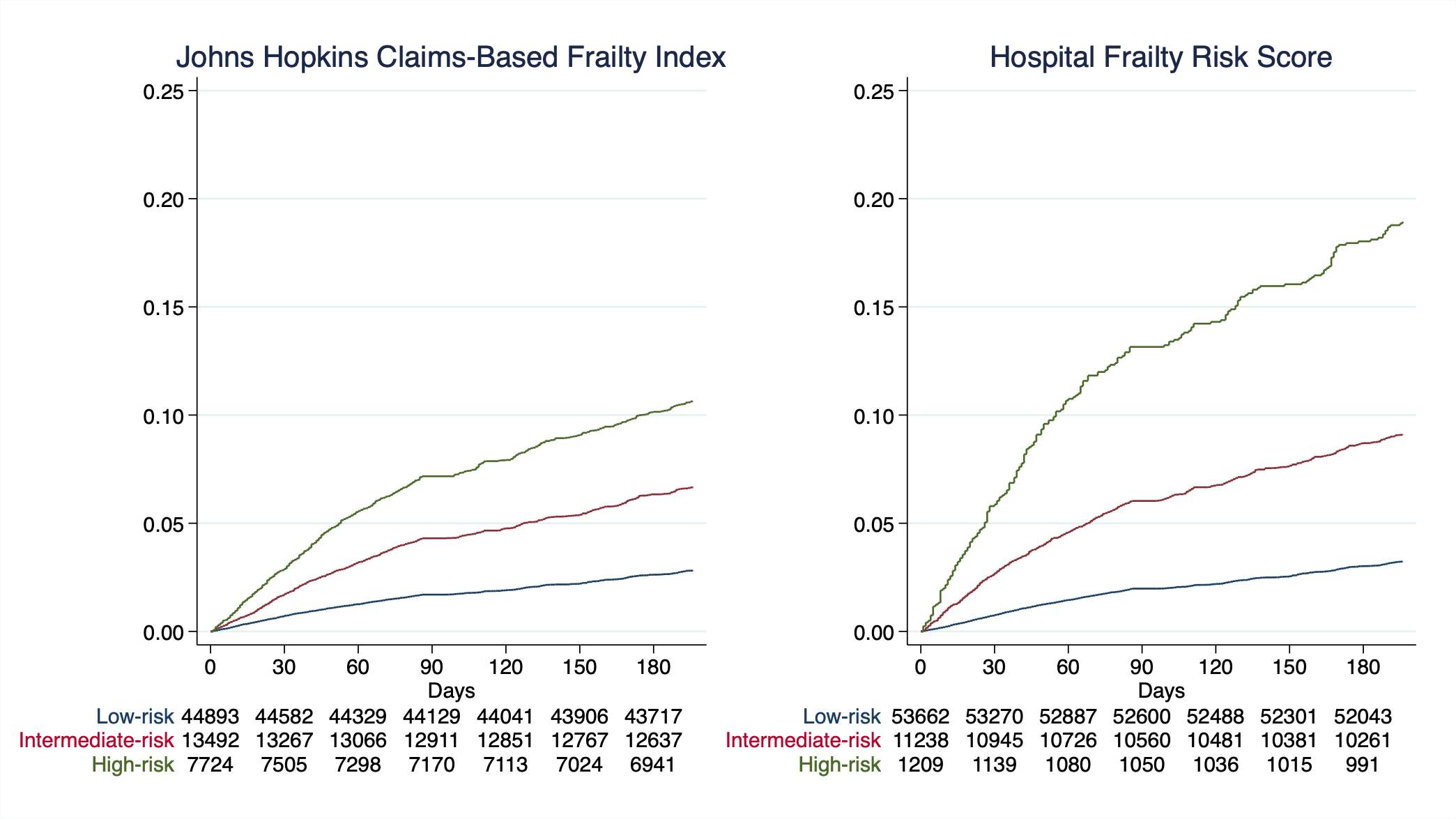

Supplement: Supplementary file 1 [file mmc1.docx]
